# 17β-Estradiol Promotes Proinflammatory and Procoagulatory Phenotype of Innate Immune Cells in the Presence of Antiphospholipid Antibodies

**DOI:** 10.3390/biomedicines8060162

**Published:** 2020-06-15

**Authors:** Gayane Manukyan, Anush Martirosyan, Ludek Slavik, Jana Ulehlova, Martin Dihel, Tomas Papajik, Eva Kriegova

**Affiliations:** 1 Laboratory of Molecular and Cellular Immunology, Institute of Molecular Biology NAS RA, Yerevan 0014, Armenia; anushmart@gmail.com; 2 Department of Immunology, Faculty of Medicine and Dentistry, Palacky University Olomouc and Faculty Hospital, 775 15 Olomouc, Czech Republic; martindihel@seznam.cz (M.D.); eva.kriegova@email.cz (E.K.); 3 Russian-Armenian (Slavonic) University, Yerevan 0051, Armenia; 4 Department of Hemato-oncology, Faculty of Medicine and Dentistry, Palacky University Olomouc and Faculty Hospital, 779 00 Olomouc, Czech Republic; ludek.slavik@fnol.cz (L.S.); jana.ulehlova@fnol.cz (J.U.); tomas.papajik@fnol.cz (T.P.)

**Keywords:** antiphospholipid syndrome, antiphospholipid antibodies, estradiol, tissue factor, monocytes, in vitro

## Abstract

Antiphospholipid syndrome (APS) is the most common cause of acquired thrombophilia and recurrent spontaneous miscarriages associated with extended persistence of antiphospholipid antibodies (aPL). How circulating aPL and high-17β-estradiol (E2) environment contribute to the pregnancy complications in APS is poorly defined. Therefore, we aimed to analyse whether E2 could be responsible for the immune cell hyperactivation in aPL- positive (lupus anticoagulant, anti-cardiolipin, anti-β2-glycoprotein) in women. For this, peripheral blood mononuclear cells (PBMCs) from 14 aPL- positive and 13 aPL- negative women were cultured in the presence or absence of E2, LPS or E2+LPS and cell immunophenotype and cytokine release were analysed. In the aPL+ group, E2 presence markedly increased the percentage of NK cells positive for CD69 (*p* < 0.05), monocytes positive for tissue factor (TF, CD142) (*p* < 0.05), and B cells expressing PD-L1 (*p* < 0.05), as well as the elevated production of IL-1β comparing to aPL- women (*p* < 0.01). Regardless of aPL positivity, E2 augmented the procoagulatory response elicited by LPS in monocytes. Our findings show the ability of E2 to promote proinflammatory and procoagulatory phenotype of innate immune cells in individuals with aPL positivity. Our data highlights the significant impact of female hormones on the activation of immune cells in the presence of aPL.

## 1. Introduction

Antiphospholipid syndrome (APS) is an acquired autoimmune condition mediated by a heterogeneous group of autoantibodies [1,2]. The presence of antiphospholipid antibodies (aPL), particularly anti-β2 glycoprotein I (anti-β2GPI), anti-cardiolipin (aCL), and lupus anticoagulant (LA), is a serological marker of APS. The syndrome covers a spectrum of clinical manifestations ranging from vascular thrombotic events to recurrent pregnancy losses and other obstetric complications [3]. Numerous epidemiological and clinical evidence has suggested a positive association between aPL and thrombotic complications in APS patients [1,2]. Experimental animal and in vitro models have supported the pathogenic role of anti-β2 GPI antibodies showing aPL- mediated activation of platelets, monocytes, endothelial cells, and increased procoagulation and fetal loss in mice [4,5]. The syndrome can be diagnosed as “primary” when there is no evidence of any underlying autoimmune processes, or “secondary” if accompanied by other autoimmune diseases, with systemic lupus erythematosus (SLE) being the most common coexisting condition [6]. APS commonly affects women in childbearing age [7], causing significant maternal morbidity and mortality in pregnant women, including recurrent miscarriages, stillbirth or premature birth, preeclampsia, eclampsia, and placental insufficiency [8].

A growing number of studies have shown that the female hormone estrogen may underlie gender bias in susceptibility to autoimmune diseases [9,10]. 17β-estradiol (E2) is the major and most potent form of estrogen where its biological role extends far beyond reproduction. In particular, E2 augments the risk of venous thromboembolism [11,12]. In physiological conditions, the E2 level rapidly increases during the first term of pregnancy and rises gradually to peak towards the term of gestation [13]. As far as normal pregnancy is a hypercoagulable state, it is not surprising that predisposed women are at an increased risk for thrombotic incidence throughout gestation [14]. In addition to naturally occurring fluctuations of hormones during life, an increase in serum E2 levels may also result from routine use of estrogen-containing medications as therapy for primary ovarian insufficiency, female hypogonadism, prevention of osteoporosis, or as an oral contraceptive [15,16].

There is growing evidence showing that immune cells are natural targets for estrogen action [17]. Estrogen has been shown to modulate innate immune cells [18], all subsets of T cells [19], and activate B cells [20]. E2 mediates its effects by binding to specific estrogen receptors via complex molecular mechanisms [21,22]. The involvement of E2 has been shown in the development or progression of several autoimmune diseases, including SLE, rheumatoid arthritis (RA), multiple sclerosis (MS) [23], and others. E2 can act in a context-dependent fashion and affect the outcome of autoimmune responses. It has been shown that estrogen decreases the severity of experimental autoimmune encephalomyelitis (EAE) and collagen-induced arthritis (CIA), while it aggravates the manifestation of SLE [17,21]. How E2 modulates immune cell activation in APS is not well understood. Thus, we aimed to analyse the impact of E2 on innate and adaptive immune cell activation in aPL positive and aPL negative women and its possible implication for the development of aPL- linked complications.

## 2. Materials and Methods

### 2.1. Patients

Two groups of middle-age non-pregnant premenopausal female volunteers were selected for the study. The patient group consisted of women positive for aCL, anti-β2GPI, and LA (aPL+ group). The control group enrolled women negative for aPL (aPL- group) with no history of thrombotic events, pregnancy loss, a concomitant autoimmune condition or family background of APS. Blood samples were taken during the follicular phase of the menstrual cycle to minimise hormonal variations. None of the donors was taking oral contraceptives or other hormonal drugs. The detailed characterisation of the patients is shown in Table 1. Blood samples were received from the University Hospital Olomouc, Czech Republic after the laboratory screening for aPL positivity (LA, aCL, anti-β2GPI) in patients suspected of having APS. Blood samples were collected into vacutainer tubes containing EDTA as an anticoagulant. The study was performed according to the Helsinki declaration after obtaining written informed consent of all participants, and it was approved by the ethics committee of the University Hospital and Palacky University Olomouc.

### 2.2. Isolation of Peripheral Blood Mononuclear Cells (PBMCs) and Culturing

Briefly, isolation of PBMCs from freshly collected blood samples was performed by density gradient centrifugation over Histopaque-1077 (Sigma-Aldrich, St. Louis, MO, USA). The mononuclear cells were recovered and washed twice in PBS. Immediately after cell separation, PBMCs (1 × 10^6^/mL) were cultured in complete RPMI 1640 medium containing 2 mM of l-Glutamine and supplemented with 10% fetal bovine serum, 100 U/mL penicillin, and 100 µg/mL streptomycin (Sigma-Aldrich) in sterile polypropylene round-bottom tubes (to reduce monocyte adherence) in a 5% CO_2_ humidified atmosphere at 37 °C. The cells were exposed to either vehicle (control), 100 ng/mL LPS (*E. coli* 0111:B4, Sigma-Aldrich), 10^−6^ M E2 (Sigma-Aldrich), or with a combination of LPS and E2. PBMCs were cultured in the absence or presence of E2 for 18 h. Afterwards, LPS was added to the corresponding tubes and incubated for a further 4 h. After 24 h culturing, cell viability was assessed by a trypan blue exclusion test and flow cytometry with 7-AAD or propidium iodide (PI) staining according to the manufacturer’s directions. The culture supernatants were harvested and stored frozen at −70 °C until further analysis.

### 2.3. Flow Cytometry Analysis

Following the cultivation period the cells were washed, aliquoted, and stained in PBS containing 0.5% BSA for the following cell-surface markers: CD3 (clone OKT3), CD4 (clone SK3), CD8 (clone SK1), CD11b (clone ICRF44), CD14 (clone M5E2), CD16 (clone 3G8), CD19 (cloneSJ25C1), CD24 (clone ML5), CD27 (clone M-T271), CD38 (HB-7), CD49d (clone 9F10), CD62L (clone DREG-56), CD69 (clone FN50), CD80 (clone 2D10), CD142 (clone NY2), HLA-DR (clone L243), PD-L1 (clone 29E.2A3), CD16/56 antibody cocktail (clones UCHT1/3G8+MEM-188) (All from BioLegend, London, UK). Isotype matched FITC, PE, PerCP-Cy5.5, Pe-Cy7, APC, and APC-Cy-7-conjugated irrelevant antibodies (BioLegend, London, UK) were used as negative controls.

A polychromatic six-colour flow cytometry analysis was performed on a Novocyte flow cytometer (ACEA Biosciences, USA). For each experiment, a minimum of 10,000 events was counted in the analysed gate. The main cell populations were identified using a sequential gating strategy after doublets’ exclusion. Cells subsets were distinguished as follows: monocytes: CD14+, T helper (Th) lymphocytes: CD3+/CD4+, T cytotoxic (Tc) lymphocytes: CD3+/CD8+, NK cells: CD3-/CD16+/CD56, B lymphocytes: CD3-/CD19+ (Figure S1). 7-AAD and PI exclusion stains were used for evaluating cell viability. Data acquisition was performed using ACEA NovoExpress software (ACEA Biosciences, USA). Flow cytometry data were analysed using the FlowJo vX0.7 software (Tree Star, Inc., San Carlos, CA, USA). The threshold for positive staining was set according to isotype controls (Figure S2). Results are expressed as a percentage and median fluorescence intensity (MFI) of the cells for each examined marker), defined as the difference between the MFI of tested cells for each examined marker and the MFI of background staining. Unimodal cell distribution was presented as MFI, while bimodal cell distribution as a percentage of positive cells.

### 2.4. Cytokine Production

Levels of interleukin (IL)-1β and tumour necrosis factor-alpha (TNF-α) in culture supernatants were quantified using commercial ELISA MAX^™^ Deluxe Set kits (BioLegend, London, UK) according to the manufacturer’s instructions. The minimum detectable cytokine concentrations were 0.5 pg/mL and 2 pg/mL for IL-1β and TNF-α, respectively.

### 2.5. Statistical Analysis

Data analysis was performed with GraphPad Prism 5.01 (GraphPad Software, San Diego, USA). All values were given as means ± standard errors of the means (SEM). Normal distribution was checked with the Shapiro–Wilk’s W test. One-way ANOVA and Wilcoxon signed-rank tests as appropriate were used to estimate the effect of treatments within aPL- and aPL+ investigated groups. The Mann–Whitney test was used for the comparisons between studied aPL- and aPL+ groups. Values of *p* < 0.05 were considered statistically significant.

## 3. Results

### 3.1. E2 Increases the Procoagulant Activity of Monocytes Isolated from aPL+ Patients

**Figure 1:**
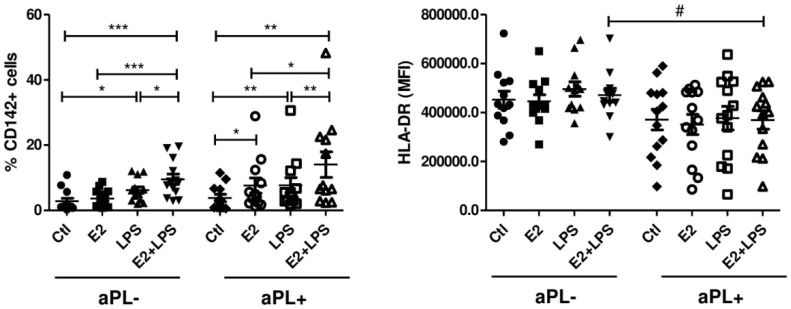
Percentage of CD142+ monocytes and HLA-DR MFI on monocytes from aPL- and aPL+ groups. PBMCs were cultured for 24 h in the absence (Ctl) and presence of E2, LPS, and E2+LPS, and analysed by flow cytometry. One-way ANOVA was used to assess statistical significance. Due to the missing data, CD142 expression on monocytes was assessed using the Wilcoxon signed-rank test. The results are shown as mean ± SEM. *^,#^ *p* < 0.05, ** *p* < 0.01, *** *p* < 0.001.

To clarify whether the levels of E2, distinctive for the first term of pregnancy, could increase the procoagulatory sensitivity of monocytes from patients with high levels of aPL, we used 10^−6^ M E2 in our in vitro model. Our results showed that E2 alone induced tissue factor (TF, CD142, or thromboplastin) response by monocytes only from aPL+ donors (*p* < 0.05) (Figure 1). TF is the primary trigger of coagulation which is inducible by aPL, thereby promoting thrombosis [5,24]. All other inducers exhibited quite similar changes in the number of cells expressing CD142 in both studied groups, and differences between aPL- and aPL+ groups were not detected (*p* > 0.05). Even though our findings did not reveal a significant modulation of TF activity in aPL- cells by E2 alone, it appeared that regardless of aPL positivity, the presence of E2 augmented the procoagulatory response elicited by proinflammatory stimuli such as LPS. Within other studied markers (CD11b, CD16, CD62L), only HLA-DR showed a tendency to the decreased expression on the cells from the aPL+ group, compared to an aPL- one. Combined stimulation of the cells with E2 and LPS exhibited a significant reduction in HLA-DR expression in the aPL+ group compared to the aPL- one (*p* < 0.05) (Figure 1). Worth mentioning that a lower expression of HLA-DR in cultured aPL+ cells was not reflective of basal levels of this molecule on circulating monocytes from aPL+ woman, which were not different from aPL- (unpublished data).

### 3.2. E2 Activates CD4+ Cells and NK Cells in aPL+ Group

**Figure 2:**
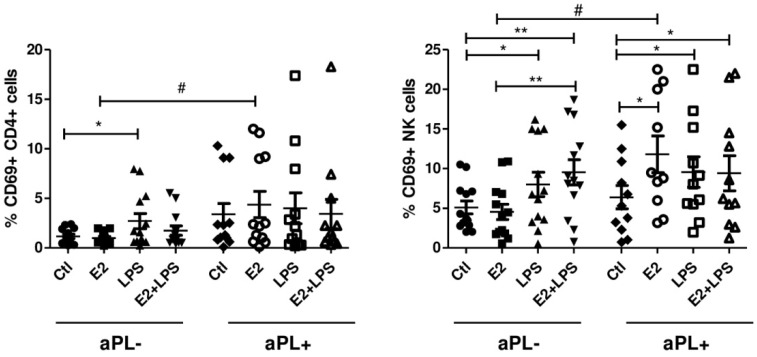
Percentage of CD69+ NK and CD4+ cells in aPL+ and aPL- groups. PBMCs were cultured for 24 h in the absence (Ctl) and presence of E2, LPS, and E2+LPS, and analysed by flow cytometry. One-way ANOVA was used to assess statistical significance. Due to the missing data, CD69 expression on NK was assessed using the Wilcoxon signed-rank test. The results are shown as mean ± SEM. *^,#^ *p* < 0.05, ** *p* < 0.01.

Cell surface expression of activation marker CD69 was used to identify activated NK cells, CD4+, and CD8+ lymphocytes. There was no evidence for the activation of cytotoxic CD8+ cells in both aPL+ and aPL- groups. In helper T cells (CD4+) from the aPL- group, LPS stimulation resulted in an increased percentage of CD69 bearing cells compared to the unstimulated cells (*p* < 0.05). Exposure of the cells with E2 resulted in a robust CD69 response from CD4+ cells in the aPL+ group compared to the aPL- one (*p* < 0.05) (Figure 2).

Similar to monocytes, the analysis of NK cells revealed E2-induced expansion of CD69+ NK cells in the aPL+ group compared to the unstimulated cells (*p* < 0.05) and those in the aPL- group (*p* < 0.05) (Figure 2). LPS and E2+LPS, as in monocytes, caused quite similar changes in the number of NK cells expressing the CD69 marker in both studied groups, while differences between the aPL- and aPL+ groups were not detected.

### 3.3. Increased Percentage of PD-L1+ B Cells in Response to E2

**Figure 3:**
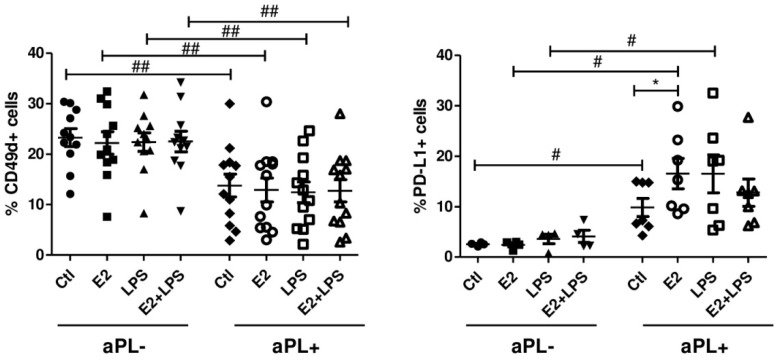
Percentage of CD49d and PD-L1+ B cells in aPL+ and aPL- groups. PBMCs were cultured for 24 h in the absence (Ctl) and presence of E2, LPS and E2+LPS, and analysed by flow cytometry. One-way ANOVA was used to assess statistical significance. The results are shown as mean ± SEM. *^,#^ *p* < 0.05, ^##^ *p* < 0.01.

Of the studied markers (CD24, CD27, CD38, CD49d, CD80), the most prominent changes in immunophenotype of B cells were received for the programmed death-ligand 1 (PD-L1) molecule (Figure 3). Stimulation of PBMCs with E2 resulted in a significant expansion of PD-L1 expressing patients-derived cells compared to unstimulated ones (*p* < 0.05) and those in the aPL- group (*p* < 0.05). The differences between aPL- and aPL+ B cells were also detected in the cells exposed to LPS (*p* < 0.05) and unstimulated cells (*p* < 0.05). When analysed integrin CD49d (VLA-4), significantly decreased the percentage of CD49d+ B cells in the aPL+ group, and they were detected after using all studied combinations of the inducers in comparison to those in the aPL- group (*p* < 0.01). Flow cytometry analysis of the cultured cells within the lymphocytes gate did not reveal any marked modulation for CD24, CD27, CD38, and CD80 within or between studied groups.

### 3.4. E2 Induces Increased Production of IL-1β by PBMCs from Patients with aPL Positivity

**Figure 4:**
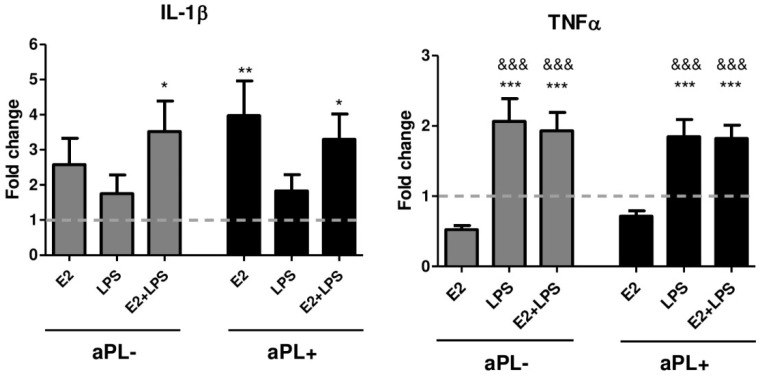
Production of IL-1β and TNF-α by PBMCs, cultured in the absence/presence of E2, LPS, E2+LPS for 24 h, was measured with ELISA. The results are expressed as fold change over the unstimulated cells. The dotted line indicates the protein levels in the supernatants of unstimulated cells. One-way ANOVA was used to assess statistical significance. The results are shown as mean ± SEM. * vs. unstimulated cells, ^&^ vs. the cells induced by E2.

To further confirm the modulating effect of E2, we measured IL-1β and TNF-α in the 24-h supernatants of PBMC cultures stimulated in the presence or absence of E2, LPS, and E2+LPS. Due to the variations in the cultured cell number between experiments, the number of cytokines released from PBMCs was normalised to the control (unstimulated) sample (Figure 4). In contrast to the results presented on monocytes (the main producers of these cytokines), no evidence of the additive or synergic effects of LPS on E2 induced cytokine production was observed. The highest levels of IL-1β were detected after E2 stimulation of PBMCs from aPL+ donors that were higher compared with unstimulated control cells (*p* < 0.01). LPS stimulation of E2-pretreated cells resulted in a higher IL-1β release from both aPL- and aPL+ groups in comparison to the unstimulated cells (*p* < 0.05).

Despite the differential responsiveness of the cells from the aPL- and aPL+ groups to the inducers, we observed nearly identical patterns of TNF-α production for both investigated groups (Figure 4). Namely, TNF-α release was up-regulated by LPS (*p* < 0.001) and E2+LPS (*p* < 0.001) when compared to the unstimulated cells in both studied groups. Worth mentioning that in contrast to IL-1β, TNF-α release in the presence of E2 was significantly lower than that induced by LPS (*p* < 0.001) and E2+LPS (*p* < 0.001), and even lower than the unstimulated control (albeit not significant).

## 4. Discussion

A predominant preference of female patients along with the tendency to manifest during the reproductive years and a variety of pregnancy-related presentations suggest that female hormonal factors play a role in the development of autoimmune APS. The most intriguing observation made in our study is the procoagulatory activation of “aPL- preconditioned” monocytes in a E2-hormonal milieu. In vitro experiments demonstrated substantial amplification of TF activity on monocytes isolated from aPL+ women and exposed to the supraphysiological concentrations of E2 [25,26]. aPL- mediated upregulation of TF is a key pathogenic event in APS [24]. Therefore, in the setting of aPL positivity, elevation of E2 may result in a prothrombotic state of monocytes favouring hypercoagulation. An association between E2 and increased risk of venous thromboembolism was first reported decades ago [27,28]. E2 induces a prothrombotic phenotype through both up-regulation of coagulation activation markers, including thrombin, and the reduction of the TF pathway inhibitor (TFPI) [29]. Yet, there is a lack of data regarding E2-mediated modulation of TF itself and additional investigations are needed to elucidate the precise mechanisms behind.

E2 exhibits bidirectional action on production of pro- and anti-inflammatory cytokines in a dose-dependent manner [30]. At low concentrations (less than 10^−8^ M), E2 potentiates LPS-induced generation of pro-inflammatory TNF-α [31], IL-6 [32], IL-1β [33], and IFN-γ [34] by PBMCs. Conversely, high concentrations of E2 (greater than 10^−6^ M) exert anti-inflammatory effects via suppression of TNF-α [35] and IL-1β [33] production in whole blood cell cultures and stimulation of IL-10 [35] and IL-1Ra [36] synthesis by PBMCs. E2 concentration used in this study was expected to have an anti-inflammatory influence on the cells. However, we have demonstrated that this concentration is able to modulate inflammatory responses in PBMCs from aPL+ donors by virtue of their enhanced IL-1β production. Even though inflammatory processes are not the most remarkable feature of APS, considerable evidence indicates a link between inflammation and both thrombotic [37] and obstetric [38] presentations. Therefore, exaggerated proinflammatory responses driven by E2 may contribute to the coagulation cascade and increased infiltration of inflammatory cells, leading to the direct or indirect placental and fetal tissue injury in women positive for aPL.

During early pregnancy, hormones, including E2, modulate immunological shifts away from inflammatory responses contributing significantly to both the pregnancy outcome and the clinical course of immune-related diseases. Particularly, pregnancy in women with MS [39,40] and RA [41,42] is accompanied by dramatic disease improvement, while patients with SLE experience significant worsening of disease activity [43,44]. In the present study, E2 levels corresponding to the early-to-mid pregnancy exerted ambivalent action on circulating immune cells. Our experiments showed an increase in B cells expressing PD-L1 from aPL+ women in the presence of E2, which can be associated with protection against autoimmune processes. Our data is in line with a study showing protection against clinical and histological signs of experimental autoimmune encephalomyelitis (EAE) through mechanisms involving PD-1 interaction with PD-L1 on B-cells in animals treated with relatively high doses of E2 (2.5 mg/60 day time-release E2 pellets) [45]. The phenomenon of entirely opposite actions of E2 on B cells has been described using low E2 doses (0.18 mg/60 day time-release E2 pellets), which resulted in the altered survival of immature B cells leading to the generation of a peripheral B cell repertoire, including autoreactive B cells that would normally be deleted [46].

Fetal death in the setting of aPL positivity has been traditionally considered to be a consequence of placental dysfunction associated with thrombosis of the utero-placental vasculature [47]. Presently, it is apparent that the whole diversity of APS-mediated features of reproductive failure could not be explained only by aPL-mediated hypercoagulable state, other non-thrombotic effects including inflammation or abnormal activity of NK cells might be involved [48,49]. Our results suggest that NK and CD4+ from aPL+ donors can be activated by E2, as seen by the increased expression of CD69. It was suggested that during early pregnancy, the migration of peripheral blood NK cells contributes to the enhancement of NK cell number in the endometrium [50]. Therefore, recruitment of circulating NK cells activated by maternal serum E2 environment may affect placentation, trophoblast invasion, and uterine spiral artery remodelling, placing aPL- positive women at higher risk.

For this study, we enrolled women of reproductive age and the main criterion of the patient’s selection was aPL positivity, which can be viewed as a limitation of the study. Similar subject selection strategy has been implemented by several researchers over the last few years, which in our view represents an important opportunity to raise awareness of potential adverse outcomes for aPL- positive pregnancies [5,51,52,53,54,55]. Potential risk of aPL- positivity underestimation was reinforced by a recent study, which reported that aPL- positive women who did not fulfil the APS criteria had comparable pregnancy outcomes, gestational period, arterial and/or venous thrombosis, compared those with confirmed APS [56]. The reasons of heightened sensitivity of individuals with high aPL titers to estradiol are largely unknown. Complex interactions of genetic characteristics of women with hormonal and environmental factors are likely modulating by epigenetic mechanisms and may lead to the aberrant activation of the innate and adaptive immune systems.

## 5. Conclusions

Collectively, our data suggests that elevated aPL titers can cause innate and adaptive immune cells to become more susceptible to E2 exposure. Consistent with this, we support existing recommendations against the estrogen/oral contraceptive use for APS patients [56,57,58], as fluctuations of estradiol levels in aPL- positive women may pose greater risk of adverse events.

## Figures and Tables

**Table 1:** A detailed demographic and clinical profile of aPL- and aPL+ donors.

	aPL- Group (Control Group)	aPL+ Group (Patients’ Group)
Number of subjects	13	14
Age (years)	34.6 (21–42)	37.5 (23–46)
Clinical records	No history of thrombotic events, pregnancy loss, familial history of APS	5/14- SLE 2/14- altered coagulation parameters 3/14- rheumatic diseases 4/14- other 2/14- pregnancy loses
LA (*n* = positive patients)	0/14	10/14
aCL (IgG) (U/ML)	<20	253.7 ± 621.8
aCL (IgM) (U/ML)	<20	69.28 ± 70.17
anti-β2GPI (IgG) (U/ML)	<20	651.0 ± 1808
anti-β2GPI (IgM) (U/ML)	<20	142.3 ± 227.3
anti-D1 β2GPI (CU/ML)	<20	175.6 ± 408

## Supplementary Materials

The following are available online at https://www.mdpi.com/2227-9059/8/6/162/s1, Figure S1: Dot plots showing gating strategy used for flow cytometric measurements of the main immune cell populations in the cultured PBMCs, Figure S2: Representative flow cytometry histogram overlays of CD142, CD69 and PD-L1 expression patterns analyzed on monocytes, CD4+, NK and B cells. All histograms represent measurements of surface markers on the cells cultured with E2. Empty gray histograms show isotype controls, blue histograms correspond to expression of markers in the cells form aPL- subjects, red to the expression of markers on the cells from aPL+ subjects Histograms were created using FlowJo software.
