# Supplementary material for: 17β-Estradiol Promotes Proinflammatory and Procoagulatory Phenotype of Innate Immune Cells in the Presence of Antiphospholipid Antibodies
Source: Biomedicines. 2020 Jun 15;8(6):162. doi: 10.3390/biomedicines8060162 (PMC7345022; doi:10.3390/biomedicines8060162)
Supplement: Supplementary file 1 [file biomedicines-08-00162-s001.pdf]

Supplementary Material

17β-estradiol promotes proinflammatory and procoagulatory phenotype of innate immune cells in the presence of antiphospholipid antibodies

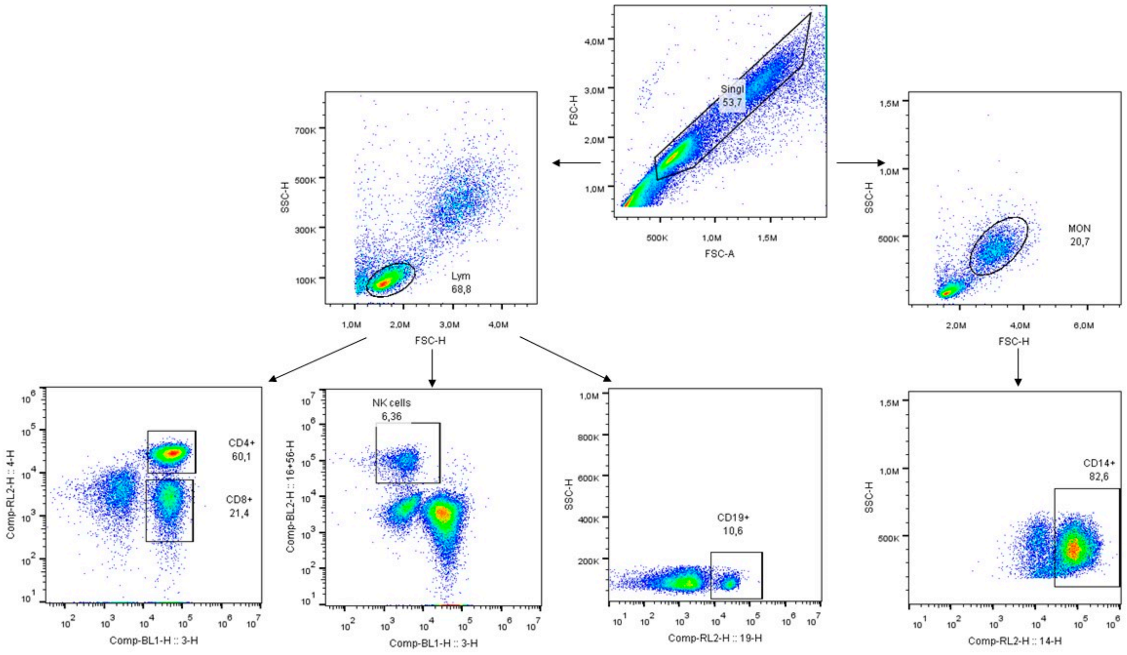

Suppl. Figure 1. Dot plots showing gating strategy used for flow cytometric measurements of the main immune cell populations in the cultured PBMCs.

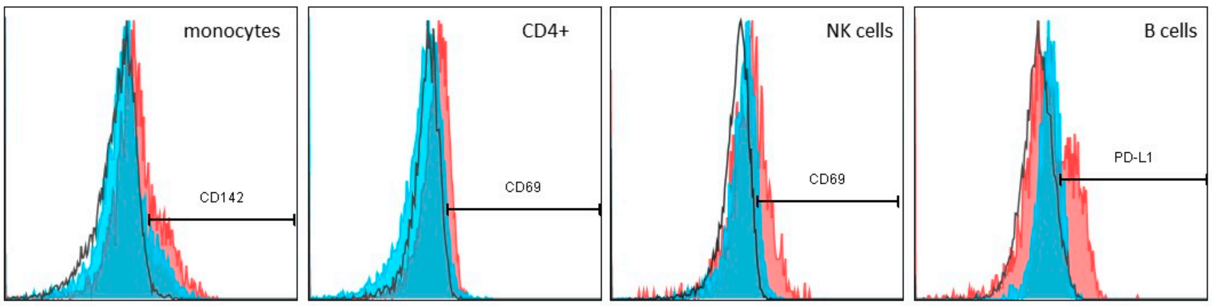

Suppl. Figure 2. Representative flow cytometry histogram overlays of CD142, CD69 and PD-L1 expression patterns analyzed on monocytes, CD4+, NK and B cells. All histograms represent measurements of surface markers on the cells cultured with E2. Empty gray histograms show isotype controls, blue histograms correspond to expression of markers in the cells form aPL- subjects, red to the expression of markers on the cells from the subjects with high aPL titers. Histograms were created using FlowJo software.
